# Acute effects of different blood flow restriction conditioning protocols on countermovement and squat jump performance

**DOI:** 10.3389/fbioe.2026.1900139

**Published:** 2026-09-18

**Authors:** Hai-Bin Yu, Ze-Feng Zheng, Yu-Hang Zhang, Jian-Zhi Lin, Bing-Kun Lai, Po-Ang Li, Chong Chen, Wei-Hsun Tai

**Affiliations:** 1 School of Physical Education, Quanzhou Normal University, Quanzhou, China; 2 Department of Physical Education, National Taiwan University of Sport, Taichung, Taiwan; 3 College of Forestry, Fujian Agriculture and Forestry University, Fuzhou, China

**Keywords:** blood flow restriction, explosive performance, movement strategy, neuromuscular fatigue, stretch-shortening cycle

## Abstract

**Background:**

This study examined the acute effects of different blood flow restriction (BFR) conditioning protocols on countermovement jump (CMJ) and squat jump (SJ) performance.

**Methods:**

Eighteen physically active males completed five experimental conditions: an unconditioned control condition (CON) and four active BFR conditioning protocols comprising 30% or 85% 1RM with 1- or 4-min recovery. CON was performed first, after which the four active conditions were randomized without replacement. CMJ and SJ order was independently randomized within each condition.

**Results:**

After false discovery rate adjustment across the biomechanical outcomes, no statistically significant condition effects remained. Likewise, no significant load, recovery-interval, or load × recovery effects were identified within the four active BFR protocols, and no jump type × condition interaction remained significant after multiplicity adjustment. Exploratory protocol-versus-CON contrasts showed differences in selected force-time variables, but these findings should be interpreted cautiously because the active protocols were preceded by a standardized warm-up whereas CON was not.

**Conclusion:**

The tested BFR conditioning protocols did not provide robust evidence of acute enhancement of explosive jump performance. The findings should be interpreted as responses to the specific conditioning protocols tested rather than as evidence of an isolated BFR effect.

## Introduction

Explosive lower-limb performance is a critical determinant of success in many athletic activities, particularly those involving sprinting, jumping, and rapid changes of direction (Papla et al., 2022; Donahue et al., 2021; Washif and Kok, 2022). Vertical jump assessments, such as the countermovement jump (CMJ) and squat jump (SJ), are widely used to evaluate neuromuscular function due to their sensitivity to changes in force production, power output, and movement strategy (Ruf et al., 2024). While both tests assess vertical force generation, they rely on distinct neuromechanical mechanisms. The CMJ incorporates the stretch-shortening cycle (SSC), enabling elastic energy storage and reflex-mediated force enhancement (Kallerud and Gleeson, 2013; Kyne and Cronin, 2025), whereas the SJ isolates concentric muscle action by eliminating the pre-stretch phase (Nishioka and Okada, 2022). As a result, comparisons between CMJ and SJ provide valuable insight into how different conditions influence reactive *versus* purely concentric force production strategies (Kozinc et al., 2022).

In recent years, blood flow restriction (BFR) training has gained considerable attention as a method to induce neuromuscular and metabolic adaptations using relatively low external loads (Wang et al., 2023). By partially occluding arterial inflow and restricting venous return, BFR creates a hypoxic intramuscular environment that accelerates fatigue, increases motor unit recruitment, and enhances metabolic stress (Zeng et al., 2025). While the chronic adaptations of BFR training are well established (Lixandrão et al., 2015), its acute effects on explosive performance remain equivocal (Wang et al., 2023; Wilk et al., 2020; Bichowska-Paweska et al., 2025). Some studies suggest that BFR may induce post-activation performance enhancement (PAPE), leading to transient improvements in power output (Zeng et al., 2025; Wei et al., 2025; Tillin and Bishop, 2009), whereas others report negligible or even detrimental effects, likely due to the interplay between potentiation and fatigue (Wang et al., 2025).

The effectiveness of BFR as a conditioning strategy may depend on several key variables, including load intensity and recovery duration (Ledergerber et al., 2026). Higher loads may induce greater potentiation but also elevate fatigue, while shorter rest intervals may limit recovery and attenuate performance benefits (Wilson et al., 2013). Conversely, longer rest periods may allow partial recovery while preserving potentiation effects (Su et al., 2025). Despite these considerations, the combined influence of load and rest interval within a BFR framework remains insufficiently understood, particularly in relation to different types of explosive tasks.

Importantly, the neuromuscular response to conditioning stimuli may be task-specific (Lin et al., 2024). The SSC mechanism in CMJ may buffer fatigue-related impairments by reutilizing elastic energy and enhancing neuromuscular efficiency (Sun et al., 2025; Tomalka, 2023), whereas the SJ, which relies entirely on voluntary concentric force production, may be more sensitive to changes in muscle contractile properties and fatigue state (Hughes et al., 2022). Therefore, examining CMJ and SJ concurrently provides a unique opportunity to distinguish whether BFR-induced effects primarily influence reactive or concentric force generation.

Accordingly, the purpose of this study was to examine the acute responses of CMJ and SJ performance to four BFR conditioning protocols that differed in external load (30% vs. 85% 1RM) and recovery interval (1 vs. 4 min). We hypothesized that the active BFR conditioning protocols would produce acute changes in explosive performance and that the magnitude of these responses would vary according to conditioning load and recovery duration.

## Materials and methods

### Participants and ethical approval

Eighteen physically active males (mean ± SD: age = 20.4 ± 1.6 years; height = 176.7 ± 5.0 cm; body mass = 70.0 ± 7.9 kg) participated in this study. All participants had previous experience with jump-based activities and reported no lower-limb musculoskeletal injuries within the previous 6 months. Before participation, participants completed a medical history questionnaire and underwent a researcher-administered interview to screen for potential contraindications to blood flow restriction (BFR) exercise. Written informed consent was obtained from all participants before participation. Participants were instructed to refrain from strenuous exercise, caffeine intake, and alcohol consumption for at least 24 h before each experimental session. The study was conducted in accordance with the Declaration of Helsinki and was approved by the Institutional Research Ethics Committee of Quanzhou Normal University (approval no. QZSYLL202561).

### Experimental design

A within-subject repeated-measures design was employed to investigate the acute effects of BFR conditioning on subsequent vertical-jump performance. Each participant completed five experimental conditions: an unconditioned control condition (CON) and four active BFR conditioning conditions comprising low-load BFR at 30% of one-repetition maximum (1RM) with either 1-min or 4-min recovery (L1 and L4, respectively), and high-load BFR at 85% 1RM with either 1-min or 4-min recovery (H1 and H4, respectively).

The CON condition was always performed first and consisted of the vertical-jump assessments without a preceding BFR conditioning stimulus. Immediately after completion of the CON condition, the participant drew one lot from the four active BFR conditions to determine the first active intervention. After completion of that intervention, the participant drew another lot from the remaining conditions to determine the next intervention. This procedure was repeated until all four active BFR conditions had been completed. Thus, the active-condition order was randomized independently for each participant without replacement. The upcoming active condition was not known until immediately before it was assigned. The order of CMJ and SJ testing was independently determined by drawing lots before each experimental condition. All 18 participants completed all five experimental conditions, and no participants were excluded from the final analysis. No experimental conditions were missing, and no protocol deviations or adverse events were reported during the experimental sessions. Two experimental sessions were conducted on the same day and were separated by an interval of at least 8 h. The first session consisted of the CON condition and two active BFR conditions, whereas the remaining two active BFR conditions were completed during the second session. Each active BFR session began with a standardized 15-min warm-up consisting of 5 min of jogging, dynamic stretching, and jump practice. Participants then rested for 1 min before performing the assigned BFR conditioning protocol. Following the conditioning protocol and the assigned recovery interval, both CMJ and SJ were assessed.

One-repetition maximum (1RM) testing was performed 2 days before the experimental sessions at the same testing facility using a standardized barbell back-squat 1RM testing procedure. The 1RM obtained during this session was subsequently used to determine the conditioning loads for the 30% and 85% 1RM BFR protocols.

### Blood flow restriction procedure and conditioning protocol

Blood flow restriction was applied bilaterally to the proximal thighs using pneumatic cuffs (Theratools, Henan, China). Individual arterial occlusion pressure (AOP) was determined using the same Theratools BFR system, which consisted of lower-limb cuffs, a hand-operated pneumatic pump, and an analog pressure gauge for direct visual monitoring of cuff pressure. The cuff was manually inflated by the operator while the pressure was continuously monitored using the integrated gauge. Individual AOP was determined according to the standard operating procedure of the device, and the target training pressure was subsequently set at 60% of the individually determined AOP (Patterson et al., 2019). AOP was reassessed before each BFR intervention, and the corresponding 60% AOP pressure was used for that intervention.

Before each active BFR condition, participants completed a standardized 15-min warm-up consisting of 5 min of jogging, dynamic stretching, and jump practice. The number and specific type of repetitions performed during the dynamic stretching and jump-practice components were not standardized or recorded; participants performed these activities according to their usual practice habits. Following the warm-up, participants rested for 1 min before beginning the conditioning protocol. Participants performed barbell back squats at either 30% or 85% of their predetermined 1RM under BFR. The low-load conditions consisted of three sets of 20, 20, and 15 repetitions, whereas the high-load conditions consisted of three sets of 2, 2, and 1 repetitions. The inter-set recovery interval was 1 min for the L1 and H1 conditions and 4 min for the L4 and H4 conditions.

Participants used their habitual standing stance during the squat exercise. Squat depth was standardized to approximately 90° of knee flexion and was supervised by the same investigator throughout the experimental procedures. No specific movement tempo was imposed, and participants performed the squat repetitions according to their habitual movement pattern. The BFR cuffs remained inflated throughout the conditioning exercise and were released immediately after completion of the squat protocol. Participants then completed the assigned recovery interval before performing the vertical-jump assessments. BFR was not applied during the CMJ or SJ assessments.

### Vertical jump testing

Vertical jump performance was assessed using two portable force platforms (T137-C, ELIGA Sports Technology Co., Ltd., Ningbo, China) sampling at 1,000 Hz. Participants stood with one foot on each force platform and kept their hands on their hips throughout the jumps to minimize the contribution of arm swing. For the CMJ, participants performed a rapid downward movement to a self-selected depth followed immediately by a maximal vertical jump. For the SJ, participants adopted a static squat position at approximately 90° of knee flexion and maintained this position for approximately 2 s before initiating the jump to minimize the contribution of the stretch-shortening cycle. Participants were instructed to jump as high as possible in both conditions.

Participants performed repeated trials until three successful trials were obtained for each jump type and experimental condition, with 60 s of rest between trials. Unsuccessful trials were excluded from analysis, and only the three successful trials were retained. The mean value of these three successful trials was calculated for subsequent statistical analysis.

### Force-time data processing

Vertical ground reaction force (vGRF) signals from the two force platforms were summed to obtain the total vertical force-time curve. The force-platform system was automatically calibrated, and body mass was determined using the same standardized weighing scale for all participants. Raw force-platform data were processed using the manufacturer’s software (ELIGA Sports Technology Co., Ltd., Ningbo, China). A 20-N force threshold was used by the software for event detection. Movement onset and subsequent movement events were automatically identified from the vGRF curve using the manufacturer’s software algorithms, and the same event-detection procedure was applied across all participants and experimental conditions.

Center-of-mass velocity was calculated by integrating the net vertical ground reaction force after accounting for body weight. The biomechanical variables were derived using conventional force-time analysis procedures implemented by the ELIGA software. Concentric power was calculated as the product of force and velocity (P = F × v), while concentric force and velocity were derived from the force-time and velocity-time curves, respectively. The following variables were obtained from the software output: jump height, concentric power, peak concentric power, peak relative power, peak concentric velocity, take-off velocity, concentric force, peak concentric force, peak relative force, push-off impulse, push-off time, and air time. For each participant and experimental condition, the mean value of the three successful trials was used for subsequent statistical analysis. The available experimental records did not retain sufficient information to independently verify whether an additional digital filtering procedure was applied before the manufacturer software generated the force-time variables. Therefore, no specific filter type, cutoff frequency, or filter order is reported.

### Statistical analysis

All statistical analyses were performed using R statistical software (version 4.5.3). Descriptive data are presented as mean ± standard deviation (SD). Linear mixed-effects models were fitted separately for each dependent variable to examine the effects of jump type and experimental condition. Jump type (CMJ vs. SJ) and condition (CON, L1, L4, H1, H4) were specified as fixed effects, and participant was included as a random intercept to account for repeated observations within participants. The models tested the main effects of jump type and condition and the jump type × condition interaction.

To specifically evaluate the four active BFR protocols, an additional factorial mixed-effects model was fitted with jump type (CMJ vs. SJ), load (30% vs. 85% 1RM), and recovery interval (1 vs. 4 min) as fixed effects and participant as a random intercept. Main effects and all two- and three-way interactions were evaluated. Estimated marginal mean differences between each active BFR protocol and CON were calculated from the fitted models, together with 95% confidence intervals (CIs). Because multiple dependent variables and multiple protocol contrasts were evaluated, the Benjamini–Hochberg false discovery rate (FDR) procedure was used to control for multiple comparisons. FDR-adjusted p values of <0.05 were considered statistically significant. All successful trials were averaged within participant and condition before inferential analysis.

## Results

Descriptive data for the assessed jump-performance and force-time variables across experimental conditions are presented in Table 1. Linear mixed-effects models were used to examine the effects of jump type and conditioning condition while accounting for repeated observations within participants. After false discovery rate adjustment across the 12 outcome variables, no statistically significant main effect of conditioning condition was observed for any outcome. The nominal condition effect for air time (Wald χ^2^ = 11.64, df = 4, unadjusted p = .020) did not remain significant after FDR adjustment (adjusted p = .243).

**TABLE 1:** Descriptive statistics of jump performance and force-time outcomes across experimental conditions.

Outcome	Jump type	CON	L1 (30%, 1 min)	L4 (30%, 4 min)	H1 (85%, 1 min)	H4 (85%, 4 min)
Air time (s)	CMJ	0.60 ± 0.04	0.62 ± 0.04	0.62 ± 0.04	0.61 ± 0.04	0.62 ± 0.04
	SJ	0.58 ± 0.04	0.59 ± 0.04	0.60 ± 0.04	0.61 ± 0.03	0.60 ± 0.03
Concentric force (N)	CMJ	1543.21 ± 186.78	1494.50 ± 170.86	1485.02 ± 167.35	1507.89 ± 177.44	1495.74 ± 130.75
	SJ	1394.20 ± 142.91	1384.86 ± 150.91	1404.02 ± 167.33	1412.95 ± 111.30	1418.68 ± 154.88
Concentric power (W)	CMJ	2303.92 ± 329.17	2289.00 ± 335.34	2248.70 ± 308.12	2310.20 ± 310.85	2280.98 ± 265.70
	SJ	1737.69 ± 227.29	1708.89 ± 230.19	1771.26 ± 338.04	1825.46 ± 251.45	1765.08 ± 269.30
Jump height (cm)	CMJ	42.29 ± 5.73	43.74 ± 5.65	43.08 ± 6.21	43.73 ± 5.27	43.70 ± 5.47
	SJ	41.03 ± 4.99	40.39 ± 4.92	42.40 ± 6.88	43.22 ± 5.19	41.35 ± 6.11
Peak concentric force (N)	CMJ	1930.30 ± 347.22	1941.87 ± 377.82	1915.94 ± 310.83	1959.76 ± 301.90	1921.88 ± 320.87
	SJ	1740.60 ± 191.62	1650.31 ± 173.00	1722.38 ± 205.41	1718.34 ± 128.16	1697.57 ± 188.74
Peak concentric force (N/kg)	CMJ	27.11 ± 4.80	27.40 ± 4.63	27.08 ± 3.99	27.61 ± 4.06	27.58 ± 4.49
	SJ	25.56 ± 2.21	23.78 ± 1.41	24.26 ± 2.46	24.08 ± 1.39	23.94 ± 1.81
Peak concentric power (W)	CMJ	4167.88 ± 621.27	4180.29 ± 585.10	4120.32 ± 541.12	4195.67 ± 622.38	4146.09 ± 529.65
	SJ	4134.43 ± 555.33	4084.24 ± 516.44	4285.31 ± 575.92	4427.27 ± 507.59	4231.64 ± 593.44
Peak concentric velocity (m/s)	CMJ	2.99 ± 0.19	3.05 ± 0.20	3.03 ± 0.22	3.05 ± 0.18	3.05 ± 0.18
	SJ	2.94 ± 0.17	2.94 ± 0.17	3.00 ± 0.23	3.03 ± 0.18	2.96 ± 0.21
Peak relative power (W/kg)	CMJ	58.34 ± 6.80	59.01 ± 6.13	58.21 ± 6.15	58.89 ± 6.51	59.36 ± 5.94
	SJ	60.67 ± 6.51	58.84 ± 5.38	60.34 ± 6.80	62.05 ± 6.35	59.65 ± 6.73
Push-off impulse (N·s)	CMJ	224.39 ± 26.31	227.61 ± 27.18	225.32 ± 23.30	227.17 ± 25.26	224.40 ± 22.02
	SJ	230.90 ± 27.52	241.44 ± 33.47	253.44 ± 41.37	244.02 ± 18.73	250.08 ± 31.05
Push-off time (s)	CMJ	0.28 ± 0.03	0.30 ± 0.03	0.30 ± 0.03	0.29 ± 0.03	0.29 ± 0.03
	SJ	0.35 ± 0.05	0.37 ± 0.04	0.39 ± 0.06	0.38 ± 0.06	0.37 ± 0.04
Take-off velocity (m/s)	CMJ	2.87 ± 0.20	2.92 ± 0.20	2.90 ± 0.22	2.92 ± 0.18	2.92 ± 0.19
	SJ	2.83 ± 0.18	2.81 ± 0.18	2.87 ± 0.24	2.90 ± 0.18	2.84 ± 0.21

Values are mean ± SD. CON, unconditioned control; L1/L4 = 30% 1RM, with 1-/4-min recovery; H1/H4 = 85% 1RM, with 1-/4-min recovery; CMJ, countermovement jump; SJ, squat jump.

Significant main effects of jump type were observed for several force-time outcomes, including concentric power, concentric force, peak concentric force, push-off time, and related outcomes; these effects reflected differences between CMJ and SJ rather than differential responses to the conditioning protocols (Table 2). No jump type × condition interaction remained statistically significant after FDR adjustment for any outcome. Thus, the present data did not provide multiplicity-adjusted evidence that the effects of the experimental conditions differed between CMJ and SJ.

**TABLE 2:** Mixed-effects model results for jump type, experimental condition, and their interaction.

Outcome	Jump type	Condition	Jump × condition
Jump height (cm)	χ^2^(1) = 1.60, q = 0.246	χ^2^(4) = 3.26, q = 0.965	χ^2^(4) = 5.84, q = 0.424
Concentric power (W)	χ^2^(1) = 117.26, q=<0.001	χ^2^(4) = 1.70, q = 0.965	χ^2^(4) = 3.18, q = 0.576
Peak concentric power (W)	χ^2^(1) = 0.12, q = 0.730	χ^2^(4) = 0.74, q = 0.965	χ^2^(4) = 7.85, q = 0.424
Peak relative power (W/kg)	χ^2^(1) = 3.86, q = 0.085	χ^2^(4) = 1.30, q = 0.965	χ^2^(4) = 5.70, q = 0.424
Peak concentric velocity (m/s)	χ^2^(1) = 2.90, q = 0.133	χ^2^(4) = 5.21, q = 0.965	χ^2^(4) = 6.17, q = 0.424
Take-off velocity (m/s)	χ^2^(1) = 1.71, q = 0.246	χ^2^(4) = 3.25, q = 0.965	χ^2^(4) = 5.52, q = 0.424
Concentric force (N)	χ^2^(1) = 23.33, q=<0.001	χ^2^(4) = 4.34, q = 0.965	χ^2^(4) = 3.55, q = 0.564
Peak concentric force (N)	χ^2^(1) = 11.24, q = 0.002	χ^2^(4) = 0.76, q = 0.965	χ^2^(4) = 2.15, q = 0.708
Peak concentric force (N/kg)	χ^2^(1) = 3.98, q = 0.085	χ^2^(4) = 0.86, q = 0.965	χ^2^(4) = 5.41, q = 0.424
Push-off impulse (N·s)	χ^2^(1) = 1.32, q = 0.273	χ^2^(4) = 0.58, q = 0.965	χ^2^(4) = 9.75, q = 0.424
Push-off time (s)	χ^2^(1) = 36.59, q=<0.001	χ^2^(4) = 3.88, q = 0.965	χ^2^(4) = 4.09, q = 0.524
Air time (s)	χ^2^(1) = 4.92, q = 0.064	χ^2^(4) = 11.64, q = 0.243	χ^2^(4) = 4.42, q = 0.524

Values are Wald χ^2^ statistics with degrees of freedom in parentheses and Benjamini–Hochberg FDR-adjusted p values (q). Participant was included as a random intercept. FDR, adjustment was applied across the 12 outcomes within each effect family.

Among the four active BFR conditions, no statistically significant main effects of load or recovery interval remained after FDR adjustment for any outcome. Likewise, no load × recovery interaction remained significant after multiplicity adjustment. No jump type × load, jump type × recovery, or jump type × load × recovery interaction remained statistically significant after FDR adjustment. These findings indicate that the tested 30% *versus* 85% 1RM loads and 1- *versus* 4-min recovery intervals did not produce robust differential effects on the assessed outcomes within the active BFR conditions.

Estimated marginal mean differences between each active BFR protocol and CON are presented in Table 3, together with 95% confidence intervals and FDR-adjusted p values. No statistically significant load, recovery-interval, or load × recovery interaction effects were identified after FDR adjustment Several contrasts showed positive point estimates for selected force-time variables, including push-off time, air time, and push-off impulse; however, these protocol-specific comparisons were treated as exploratory in view of the multiple outcomes and contrasts examined.

**TABLE 3:** Mixed-effects factorial analysis of the four active BFR conditioning protocols.

Outcome	Jump type	Load	Recovery	Jump × load	Jump × recovery	Load × recovery	Jump × load × recovery
Jump height (cm)	χ^2^(1) = 11.68, q=<0.001	χ^2^(1) = 0.00, q = 0.990	χ^2^(1) = 0.45, q = 0.896	χ^2^(1) = 4.20, q = 0.095	χ^2^(1) = 3.71, q = 0.187	χ^2^(1) = 0.21, q = 0.954	χ^2^(1) = 5.28, q = 0.062
Concentric power (W)	χ^2^(1) = 122.60, q=<0.001	χ^2^(1) = 0.16, q = 0.990	χ^2^(1) = 0.59, q = 0.896	χ^2^(1) = 1.66, q = 0.340	χ^2^(1) = 1.92, q = 0.249	χ^2^(1) = 0.02, q = 0.954	χ^2^(1) = 1.63, q = 0.346
Peak concentric power (W)	χ^2^(1) = 1.11, q = 0.318	χ^2^(1) = 0.03, q = 0.990	χ^2^(1) = 0.43, q = 0.896	χ^2^(1) = 6.46, q = 0.095	χ^2^(1) = 4.10, q = 0.187	χ^2^(1) = 0.01, q = 0.954	χ^2^(1) = 4.99, q = 0.062
Peak relative power (W/kg)	χ^2^(1) = 0.02, q = 0.886	χ^2^(1) = 0.01, q = 0.990	χ^2^(1) = 0.47, q = 0.896	χ^2^(1) = 4.12, q = 0.095	χ^2^(1) = 1.96, q = 0.249	χ^2^(1) = 0.59, q = 0.954	χ^2^(1) = 4.96, q = 0.062
Peak concentric velocity (m/s)	χ^2^(1) = 12.62, q=<0.001	χ^2^(1) = 0.02, q = 0.990	χ^2^(1) = 0.62, q = 0.896	χ^2^(1) = 4.38, q = 0.095	χ^2^(1) = 3.67, q = 0.187	χ^2^(1) = 0.41, q = 0.954	χ^2^(1) = 5.79, q = 0.062
Take-off velocity (m/s)	χ^2^(1) = 11.47, q=<0.001	χ^2^(1) = 0.00, q = 0.990	χ^2^(1) = 0.48, q = 0.896	χ^2^(1) = 3.93, q = 0.095	χ^2^(1) = 3.47, q = 0.187	χ^2^(1) = 0.19, q = 0.954	χ^2^(1) = 5.04, q = 0.062
Concentric force (N)	χ^2^(1) = 13.96, q=<0.001	χ^2^(1) = 0.21, q = 0.990	χ^2^(1) = 0.10, q = 0.896	χ^2^(1) = 0.13, q = 0.789	χ^2^(1) = 0.48, q = 0.535	χ^2^(1) = 0.00, q = 0.954	χ^2^(1) = 0.03, q = 0.854
Peak concentric force (N)	χ^2^(1) = 30.19, q=<0.001	χ^2^(1) = 0.11, q = 0.990	χ^2^(1) = 0.24, q = 0.896	χ^2^(1) = 0.45, q = 0.672	χ^2^(1) = 1.71, q = 0.255	χ^2^(1) = 0.03, q = 0.954	χ^2^(1) = 0.58, q = 0.595
Peak concentric force (N/kg)	χ^2^(1) = 24.27, q=<0.001	χ^2^(1) = 0.08, q = 0.990	χ^2^(1) = 0.20, q = 0.896	χ^2^(1) = 0.01, q = 0.924	χ^2^(1) = 0.60, q = 0.525	χ^2^(1) = 0.08, q = 0.954	χ^2^(1) = 0.39, q = 0.636
Push-off impulse (N·s)	χ^2^(1) = 5.48, q = 0.023	χ^2^(1) = 0.01, q = 0.990	χ^2^(1) = 0.15, q = 0.896	χ^2^(1) = 0.13, q = 0.789	χ^2^(1) = 2.93, q = 0.209	χ^2^(1) = 0.00, q = 0.954	χ^2^(1) = 0.21, q = 0.702
Push-off time (s)	χ^2^(1) = 40.57, q=<0.001	χ^2^(1) = 0.14, q = 0.990	χ^2^(1) = 0.00, q = 0.959	χ^2^(1) = 0.86, q = 0.532	χ^2^(1) = 2.42, q = 0.240	χ^2^(1) = 0.13, q = 0.954	χ^2^(1) = 1.40, q = 0.356
Air time (s)	χ^2^(1) = 13.26, q=<0.001	χ^2^(1) = 0.05, q = 0.990	χ^2^(1) = 0.00, q = 0.959	χ^2^(1) = 4.33, q = 0.095	χ^2^(1) = 0.14, q = 0.711	χ^2^(1) = 0.29, q = 0.954	χ^2^(1) = 2.28, q = 0.262

Values are Wald χ^2^ statistics with degrees of freedom in parentheses and Benjamini–Hochberg FDR-adjusted p values (q). Participant was included as a random intercept. The analysis included jump type, load (30% vs. 85% 1RM), recovery (1 vs. 4 min), and all interactions among the four active BFR, conditions.

## Discussion

The present study examined the acute effects of different BFR conditioning protocols on subsequent CMJ and SJ performance, with particular attention to conditioning load and recovery interval. The revised analyses provide no multiplicity-adjusted evidence that the active BFR conditioning protocols produced systematic improvements in jump performance or force-time outcomes. In the factorial analysis restricted to the four active protocols, no statistically significant effects were identified for conditioning load, recovery interval, or their interaction after false discovery rate (FDR) adjustment. Likewise, no evidence supported differential responses between CMJ and SJ across experimental conditions. These findings therefore do not support a robust acute performance-enhancing effect of the specific BFR conditioning protocols examined in the present study.

Contrary to the initial hypothesis, the tested BFR protocols did not produce the performance enhancement commonly associated with post-activation performance enhancement (PAPE). Previous research indicates that potentiation following conditioning contractions may involve increased motor-unit recruitment, phosphorylation of myosin regulatory light chains, and enhanced neural drive (Tillin and Bishop, 2009; Blazevich and Babault, 2019). However, the expression of PAPE depends on the balance between potentiation and fatigue, as well as on the characteristics of the conditioning stimulus and recovery period (Corvino et al., 2022). The absence of clear improvements in jump height and power variables in the present study may therefore reflect an insufficient net potentiation response. Given the hypoxic and metabolically demanding nature of BFR exercise, fatigue may have offset any transient potentiation that might otherwise have been expressed (Boullosa et al., 2018; Wang et al., 2023). However, because neuromuscular activation, metabolic stress, muscle oxygenation, and peripheral fatigue were not directly measured, this interpretation should be regarded as a plausible mechanism rather than a direct finding of the present study.

Although the primary performance outcomes did not show robust improvements, selected force-time variables differed across experimental conditions. Previous work has suggested that force-time characteristics can provide additional information about acute neuromuscular responses that may not be captured by jump height alone (Gathercole et al., 2015). Changes in push-off impulse and push-off time may reflect alterations in the organization of force production during the jump, while previous BFR research has also reported changes in jump-related performance characteristics without clear improvements in overall jump performance (Horiuchi et al., 2018). Such changes may represent adjustments in movement execution under the physiological demands imposed by the conditioning stimulus. Nevertheless, because the revised analyses did not demonstrate robust effects across the full set of outcomes after FDR correction, these findings should be interpreted as exploratory rather than evidence of a consistent compensatory strategy.

An important consideration is that the present study evaluated specific BFR conditioning protocols rather than the isolated effect of BFR. Because all active conditions combined BFR with barbell back-squat exercise and no matched non-BFR conditioning condition was included, the independent contribution of BFR cannot be determined. Thus, protocol-versus-CON differences should be interpreted as responses to the tested conditioning packages rather than definitive BFR-specific effects. The present findings indicate that the specific combinations of load, repetition structure, BFR, and recovery examined here did not produce robust differential responses. CMJ and SJ differ biomechanically, with CMJ involving the stretch-shortening cycle (SSC) and SJ emphasizing concentric force production (Bobbert et al., 1996; Van Hooren and Zolotarjova, 2017). These differences may contribute to distinct force-time responses to fatigue or conditioning stimuli (Berger and d'Avella, 2014; Horita et al., 1996). However, the absence of a significant jump type × condition interaction does not support the conclusion that SJ was more sensitive to BFR conditioning than CMJ. Thus, the previously proposed SSC-related buffering effect in CMJ should be regarded as a plausible hypothesis rather than an established mechanism.

From a practical perspective, the tested BFR protocols do not appear to provide a reliable acute strategy for enhancing subsequent explosive performance. Although selected force-time variables differed across conditions, no robust load, recovery, or jump type × condition effects were identified after multiplicity adjustment. Previous studies have likewise reported limited effects of BFR on jump performance, whereas other work has observed performance benefits under specific BFR conditions (Horiuchi et al., 2018; Hu et al., 2025; Zhou et al., 2024). These differences highlight the importance of conditioning protocol, exercise characteristics, recovery, and outcome selection when interpreting acute BFR responses.

### Limitations

Several limitations should be considered when interpreting the present findings. First, the sample consisted exclusively of physically active males, and only acute responses were examined, limiting generalizability to other populations and chronic BFR adaptations. Second, no load- and volume-matched non-BFR condition was included, while the 30% and 85% 1RM protocols also differed substantially in repetition volume (55 vs. 5 repetitions). Thus, the independent effects of BFR, resistance load, and conditioning volume cannot be isolated. Third, CON was performed without the standardized warm-up used before the active conditions, and the two daily sessions were separated by 8 h. Because physiological carryover was not directly assessed and intervention sequences were not retained, residual period or carryover effects cannot be completely excluded. Finally, multiple outcomes were examined without a prespecified primary endpoint, although FDR adjustment and model-based effect estimates with 95% confidence intervals were applied. Physiological mechanisms were also not directly measured; therefore, mechanistic explanations should be regarded as hypotheses for future investigation.

## Conclusion

The tested BFR conditioning protocols did not produce multiplicity-adjusted evidence of acute improvements in subsequent CMJ or SJ performance. Within the four active protocols, no robust effects of conditioning load, recovery interval, or their interaction were identified after FDR adjustment. Although some protocol-versus-CON differences were observed in selected force-time variables, these findings should be interpreted cautiously because the active protocols were preceded by a standardized warm-up whereas CON was not, and because the study design did not include a matched non-BFR conditioning control. Furthermore, the present data do not support the conclusion that SJ is more responsive to BFR conditioning than CMJ. Overall, the findings indicate that the specific BFR conditioning packages examined in this study did not reliably enhance acute explosive performance, while highlighting the need for future studies with matched control conditions, prespecified primary outcomes, and direct physiological measurements.

## Data Availability

The original contributions presented in the study are included in the article/Supplementary Material, further inquiries can be directed to the corresponding authors.

## References

[B1] Berger D. J. d'Avella A. (2014). Effective force control by muscle synergies. Front. Computational Neuroscience 8, 46. 10.3389/fncom.2014.00046 PMC4029017 24860489

[B2] Bichowska-Paweska M. Fostiak K. Gawel D. Trybulski R. Alexe D. I. Szura E. (2025). The acute effect of blood flow restriction or ischemia on countermovement jump performance. J. Hum. Sport Exerc. 20 (2), 658–670. 10.55860/1wmw9h79

[B3] Blazevich A. J. Babault N. (2019). Post-activation potentiation *versus* post-activation performance enhancement in humans: historical perspective, underlying mechanisms, and current issues. Front. Physiology 10, 1359. 10.3389/fphys.2019.01359 31736781 PMC6838751

[B4] Bobbert M. F. Gerritsen K. G. M. Litjens M. C. A. Soest A. J. V. (1996). Why is countermovement jump height greater than squat jump height? Med. and Sci. Sports and Exerc. 28 (11), 1402–1412. 10.1097/00005768-199611000-00009 8933491

[B5] Boullosa D. Rosso S. D. Behm D. G. Foster C. (2018). Post-activation potentiation (PAP) in endurance sports: a review. Eur. Journal Sport Science 18 (5), 595–610. 10.1080/17461391.2018.1438519 29490594

[B7] Corvino R. B. Scheffer D. da L. Penteado Dos Santos R. Latini A. Oliveira A. S. Caputo F. (2022). Muscle fatigue is attenuated when applying intermittent compared with continuous blood flow restriction during endurance cycling. Int. J. Sports Physiology Perform. 17 (7), 1126–1131. 10.1123/ijspp.2021-0523 35551112

[B8] Donahue T. Paul S. J. W. Williams C. C. Hill C. M. Garner J. C. (2021). Comparison of countermovement and squat jumps performance in recreationally trained males. Int. J. Exercise Science 14 (1), 462–472. 10.70252/FXQB8485 PMC8136569 34055142

[B9] Gathercole R. Sporer B. Stellingwerff T. Sleivert G. (2015). Alternative countermovement-jump analysis to quantify acute neuromuscular fatigue. Int. J. Sports Physiology Performance 10 (1), 84–92. 10.1123/ijspp.2013-0413 24912201

[B10] Horita T. Komi P. V. Nicol C. Kyröläinen H. (1996). Stretch shortening cycle fatigue: interactions among joint stiness, reflex, and muscle mechanical performance in the drop jump. Eur. J. Appl. Physiology Occup. Phys. 73 (5), 393–403. 10.1007/BF00334415 8803498

[B11] Horiuchi M. Endo J. Sato T. Okita K. (2018). Jump training with blood flow restriction has no effect on jump performance. Biol. Sport 35 (4), 343–348. 10.5114/biolsport.2018.78053 30765919 PMC6358527

[B12] Hu C. Du N. Li Y. Girard O. Mei T. (2025). Enhancing jump performance through blood flow restriction squat exercise: a muscle synergy analysis using wavelet packet transformation. J. Sports Sci. and Med. 24 (3), 578–588. 10.52082/jssm.2025.578 40933326 PMC12418193

[B13] Hughes S. Warmenhoven J. Haff G. G. Chapman D. W. Nimphius S. (2022). Countermovement jump and squat jump force-time curve analysis in control and fatigue conditions. J. Strength and Cond. Res. 36 (10), 2752–2761. 10.1519/JSC.0000000000003955 35687846 PMC9488939

[B14] Kallerud H. Gleeson N. (2013). Effects of stretching on performances involving stretch-shortening cycles. Sports Medicine 43 (8), 733–750. 10.1007/s40279-013-0053-x 23681447

[B15] Kozinc Ž. Žitnik J. Smajla D. Šarabon N. (2022). The difference between squat jump and countermovement jump in 770 male and female participants from different sports. Eur. J. Sport Sci. 22 (7), 985–993. 10.1080/17461391.2021.1936654 34075858

[B16] Kyne H. Cronin J. B. (2025). Beyond jump height: a comparison of concentric variables in the squat jump, countermovement jump and drop jump for athletic profiling. Sports 13 (11), 379. 10.3390/sports13110379 41295762 PMC12656558

[B17] Ledergerber R. Ritsche P. Lichtenstein E. Prechtl L. Faude O. Keller M. (2026). Effects of low‐load blood‐flow restriction training *versus* high‐load resistance training on neuromuscular performance and neuromuscular activation. Scand. J. Med. and Sci. Sports 36 (1), e70203. 10.1111/sms.70203 41502043 PMC12780320

[B18] Lin Y.-T. Wong C.-M. Chen Y.-C. Chen Y. Hwang I.-S. (2024). Differential training benefits and motor unit remodeling in wrist force precision tasks following high and low load blood flow restriction exercises under volume-matched conditions. J. NeuroEngineering Rehabilitation 21 (1), 123. 10.1186/s12984-024-01419-5 39030574 PMC11264616

[B19] Lixandrão M. E. Ugrinowitsch C. Laurentino G. Libardi C. A. Aihara A. Y. Cardoso F. N. (2015). Effects of exercise intensity and occlusion pressure after 12 weeks of resistance training with blood-flow restriction. Eur. J. Applied Phys. 115 (12), 2471–2480. 10.1007/s00421-015-3253-2 26323350

[B20] Nishioka T. Okada J. (2022). Associations of maximum and reactive strength indicators with force–velocity profiles obtained from squat jump and countermovement jump. PloS One 17 (10), e0276681. 10.1371/journal.pone.0276681 36269787 PMC9586393

[B21] Papla M. Latocha A. Grzyb W. Gołaś A. (2022). Relationship between lower limb power output, sprint and change of direction performance in soccer players. Baltic J. Health Phys. Activity 14 (3), 3. 10.29359/bjhpa.14.3.03

[B22] Patterson S. D. Hughes L. Warmington S. Burr J. Scott B. R. Owens J. (2019). Blood flow restriction exercise: considerations of methodology, application, and safety. Front. Phys. 10, 533. 10.3389/fphys.2019.00533 31156448 PMC6530612

[B23] Ruf L. Altmann S. Müller K. Rehborn A. Schindler F. Woll A. (2024). Concurrent validity of countermovement and squat jump height assessed with a contact mat and force platform in professional soccer players. Front. Sports Act. Living 6, 1437230. 10.3389/fspor.2024.1437230 39045566 PMC11263071

[B24] Su C. Zhang Z. Liang B. Zhou S. Long X. (2025). Effects of blood flow restriction combined with high-load training on muscle strength and sports performance in athletes: a systematic review and meta-analysis. Front. Physiology 16, 1603568. 10.3389/fphys.2025.1603568 40671707 PMC12263612

[B25] Sun J. Sun J. Shaharudin S. Zhang Q. (2025). Effects of plyometrics training on lower limb strength, power, agility, and body composition in athletically trained adults: systematic review and meta-analysis. Sci. Rep. 15 (1), 34146. 10.1038/s41598-025-10652-4 41034241 PMC12489078

[B26] Tillin N. A. Bishop D. (2009). Factors modulating post-activation potentiation and its effect on performance of subsequent explosive activities. Sports Medicine 39 (2), 147–166. 10.2165/00007256-200939020-00004 19203135

[B27] Tomalka A. (2023). Eccentric muscle contractions: from single muscle fibre to whole muscle mechanics. Pflügers Archiv-European J. Physiology 475 (4), 421–435. 10.1007/s00424-023-02794-z 36790515 PMC10011336

[B28] Van Hooren B. Zolotarjova J. (2017). The difference between countermovement and squat jump performances: a review of underlying mechanisms with practical applications. J. Strength and Cond. Res. 31 (7), 2011–2020. 10.1519/JSC.0000000000001913 28640774

[B29] Wang X. Qin X.-M. Ji S. Dong D. (2023). Effects of resistance training with blood flow restriction on explosive power of lower limbs: a systematic review and meta-analysis. J. Human Kinetics 89, 259–268. 10.5114/jhk/168308 38053964 PMC10694717

[B30] Wang J. Xu J. Liu H. Jiang L. (2025). The immediate effects of blood flow restriction training on upper limb muscle strength and fatigue level: a meta-analysis. Front. Physiology 16, 1521145. 10.3389/fphys.2025.1521145 40666112 PMC12259574

[B31] Washif J. A. Kok L.-Y. (2022). Relationships between vertical jump metrics and sprint performance, and qualities that distinguish between faster and slower sprinters. J. Sci. Sport Exerc. 4 (2), 135–144. 10.1007/s42978-021-00122-4

[B32] Wei X. Zheng X. Tong J. Zhao Z. Zheng H. Wang T. (2025). Effects of blood flow restriction combined with low resistance training on post-activation performance enhancement in male volleyball players. BMC Sports Sci. Med. Rehabilitation 17 (1), 353. 10.1186/s13102-025-01422-w PMC12645768 41287005

[B33] Wilk M. Krzysztofik M. Filip A. Szkudlarek A. Lockie R. G. Zajac A. (2020). Does post-activation performance enhancement occur dring the bench press exercise under blood flow restriction? Int. J. Environ. Res. Public Health 17 (11), 3752. 10.3390/ijerph17113752 32466396 PMC7312758

[B34] Wilson J. M. Duncan N. M. Marin P. J. Brown L. E. Loenneke J. P. Wilson S. M. C. (2013). Meta-analysis of postactivation potentiation and power: effects of conditioning activity, volume, gender, rest periods, and training status. J. Strength and Cond. Res. 27 (3), 854–859. 10.1519/JSC.0b013e31825c2bdb 22580978

[B35] Zeng N. Liu H. Wang J. Jiang L. (2025). The effects of blood flow restriction training on post activation potentiation and fatigue level: systematic review with meta-analysis. Front. Physiology 16, 1558008. 10.3389/fphys.2025.1558008 40276367 PMC12018386

[B36] Zhou L. Tan Y. Gan J. Li C. Bao D. Zhou J. (2024). Complex training with blood flow restriction increases power output and bar velocity during half-squat jump: a pilot randomized controlled study. Front. Physiology 15, 1368917. 10.3389/fphys.2024.1368917 38883184 PMC11177751

